# HPV16-E2 induces prophase arrest and activates the cellular DNA damage response *in vitro* and in precursor lesions of cervical carcinoma

**DOI:** 10.18632/oncotarget.5512

**Published:** 2015-10-14

**Authors:** Yuezhen Xue, Shen Yon Toh, Pingping He, Thimothy Lim, Diana Lim, Chai Ling Pang, Jean-Pierre Abastado, Françoise Thierry

**Affiliations:** ^1^ Institute of Medical Biology, A*STAR, Singapore; ^2^ Department of Gynaecological Oncology, KK Women's and Children's Hospital, Singapore; ^3^ Department of Pathology, National University Hospital, Singapore; ^4^ Singapore Immunology Network, A*STAR, Singapore; ^5^ Current address: p53 Laboratory, A*STAR, Singapore

**Keywords:** HPV16-E2, DNA damage response, cell cycle, prophase, cervical intraepithelial neoplasia

## Abstract

Cervical intraepithelial neoplasia (CIN) is caused by human papillomavirus (HPV) infection and is the precursor to cervical carcinoma. The completion of the HPV productive life cycle depends on the expression of viral proteins which further determines the severity of the cervical neoplasia. Initiation of the viral productive replication requires expression of the E2 viral protein that cooperates with the E1 viral DNA helicase. A decrease in the viral DNA replication ability and increase in the severity of cervical neoplasia is accompanied by simultaneous elevated expression of E6 and E7 oncoproteins. Here we reveal a novel and important role for the HPV16-E2 protein in controlling host cell cycle during malignant transformation. We showed that cells expressing HPV16-E2 *in vitro* are arrested in prophase alongside activation of a sustained DDR signal. We uncovered evidence that HPV16-E2 protein is present *in vivo* in cells that express both mitotic and DDR signals specifically in CIN3 lesions, immediate precursors of cancer, suggesting that E2 may be one of the drivers of genomic instability and carcinogenesis *in vivo*.

## INTRODUCTION

Cervical cancer is the second most common cause of cancer-related death in women worldwide and arises through a progressive graded dysplasia of the cervical epithelium, pathologically termed cervical intraepithelial neoplasia (CIN) [1, 2]. Persistent HPV infection with high-risk viral subtypes is now well established as a cause of both CIN and cervical cancer, but the molecular events underlying carcinogenic progression are still poorly understood [3]. The HPV life cycle is divided into two main phases; the productive phase and the abortive phase. The productive phase usually prevails during the milder stages of cervical dysplasia, graded CIN1 or CIN2, where viral genomes are maintained as low-copy-number episomes in cells of the lower epithelial layers, becoming amplified as the infected cell migrates towards the epithelial surface during cellular differentiation [4, 5]. In the abortive phase, HPV presents as persistent infection with low copy numbers of HPV DNA detected in cells.

Viral protein E2 is required for the initiation of viral DNA replication, in cooperation with the E1 DNA helicase. We recently demonstrated that precursor lesions of HPV16-associated cervical cancers express high levels of E2, before high levels of the viral oncogene E7. E2 expression is evident in the intermediate and/or upper layers of low grade CIN, and occurs alongside initiation of viral replication and productive amplification of the HPV genome [6]. The E7 protein is expressed in the basal and para-basal layers of low grade CIN, as well as in all layers in severe CIN3 and squamous cell carcinoma (SCC), where the extent of its expression closely correlates with malignant progression [7]. The viral products detected in the infected cervix depend upon how complete the productive life cycle is, which in turn determines the severity of the CIN. In CIN3, which is the immediate precursor of cervical carcinoma, cells appear immature and viral replication is low. While the majority of low grade CIN can regress together with viral clearance, 30–50% of CIN3 lesions will ultimately progress to invasive cervical cancer with integration of part of the viral genome in the cellular genome [8].

Recent studies in human precancerous lesions suggest that there is activation of the DNA damage checkpoint and high levels of genomic instability in the cells [9, 10]. It has also been proposed that HPV hijacks the DNA damage response (DDR) machinery of the host cell to support its own genome amplification, rather than relying on the cellular replication machinery [11, 12]. However, the relevance and mechanisms of these processes during progression towards cervical carcinoma remain unclear. We hypothesized that HPV-E2 protein might also be involved in initiating the DNA damage response as it is part of the viral DNA replication process and is expressed in precursors of cancer. E2 from the high risk HPV16 and HPV18 types can directly induce cell cycle arrest during mitosis and lead to genomic instability [13, 14]. Recent reports have also linked normal mitotic entry of non-infected cell with the induction of a DDR signal [15–17]. Therefore, in this study we asked whether HPV16-E2 was involved in induction of the DDR after host cell entry into M phase *in vitro* and whether that could also be demonstrated *in vivo*, in patient samples.

We found that E2 expression *in vitro* induces cell cycle arrest in prophase and promotes sustained activation of a DDR signal. In patient samples of CIN3 lesions, E2 and the E7 surrogate marker p16 were co-expressed specifically in the intermediate and upper layers in a subset of infected tissues with an increased population of prophase cells. In parallel, we detected activation of the DDR signal in prophase cells in these lesions, which similarly co-expressed E2 and the E7 surrogate marker p16, and exhibited low levels of viral DNA replication.

## RESULTS

### HPV16-E2 protein induces cell cycle arrest in prophase

We previously reported that high-risk HPV-E2 protein can induce cell cycle arrest during mitosis in various cell types, even in the absence of other viral proteins [13]. In this study we further characterized the HPV-16E2 induced mitotic arrest in cervical carcinoma cells *in vitro* where E2 is expressed via adenoviral transduction. We used SiHa cervical carcinoma cell line positive for HPV16 to explore the role of E2 in cell cycle progression. The cells were synchronized by double thymidine block and infected with GFP, GFP-16E2, GFP-DBD or GFP-TAD recombinant adenoviruses at multiplicity of infection (m.o.i) of 50 (these latter two constructs containing the C-terminal DNA binding domain - DBD or the N-terminal transactivation domain - TAD of the high risk HPV16 E2 protein) [18]. The DBD of high risk HPV E2 does not have E2 transactivation function and can bind to the endogenous E6E7 promoter to inhibit E6E7 transcription as well as the full length E2 protein. In contrast TAD exhibits most of the other functions of E2. Consequently in SiHa cells infected with the GFP-16E2 recombinant adenovirus, most of E6E7 transcription is repressed and the transduced E2 is highly expressed (E2), while in TAD expressing cells, E2, E6 and E7 are expressed together (E2 + E6E7) and in DBD expressing cells, E2 is not expressed with simultaneous inhibition of E6 and E7 (−). In the control GFP infected SiHa cells, endogenous E6E7 is highly expressed (E6E7) in the absence of any endogenous E2 expression [19].

**Figure 1:**
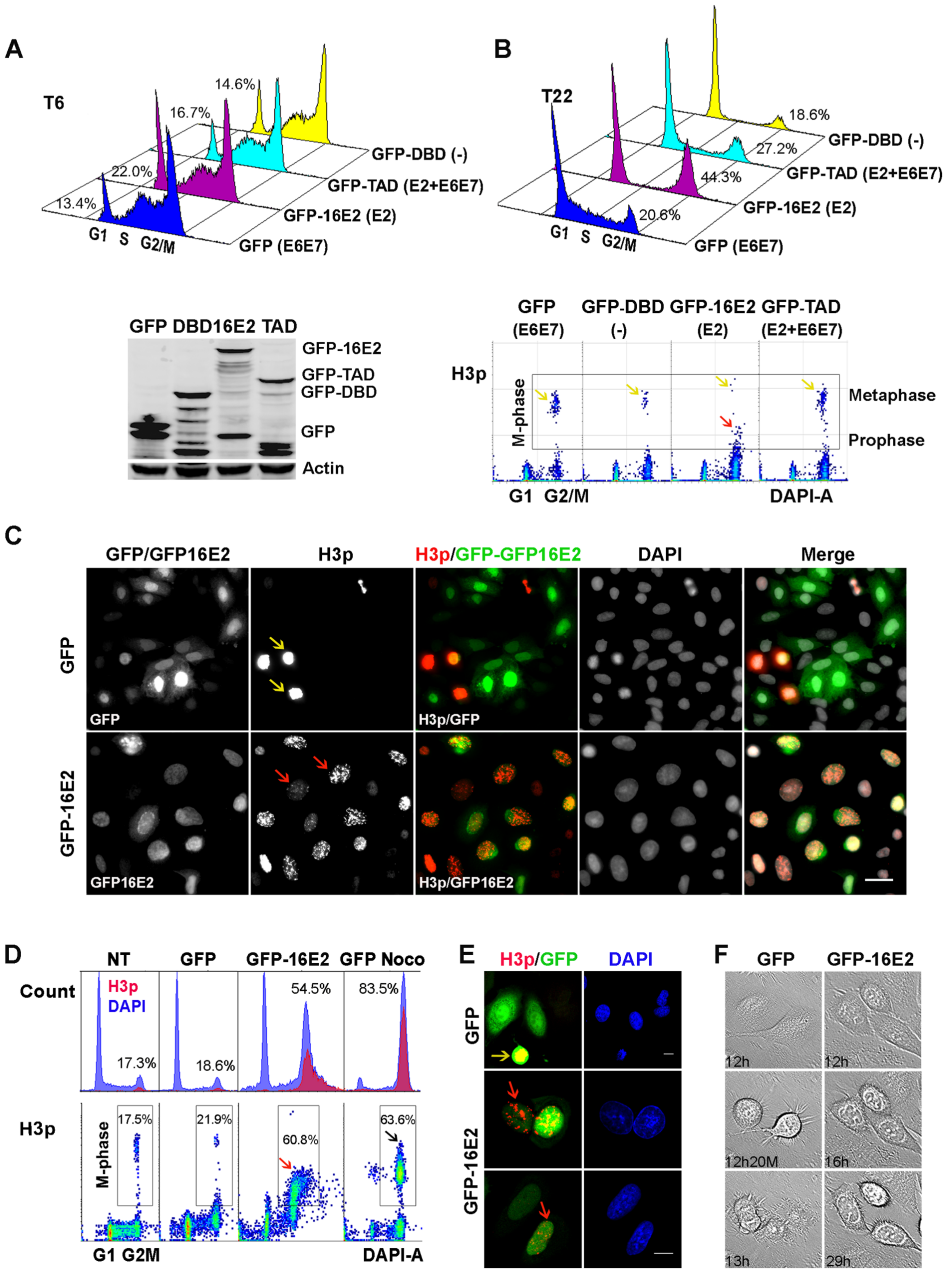
SiHa and A549 cells that express E2 are arrested in prophase **A.** Flow cytometric analysis of cell cycle distribution of SiHa cells infected with recombinant adenoviruses expressing GFP, GFP-16E2, GFP-DBD or GFP-TAD of HPV16 E2, 6 hours post thymidine release and their protein levels analysed at the same time points by Western blot (lower panel). **B.** As in (A) on cells harvested 22 hours post thymidine release. H3p labeling density plots corresponding to the same samples are shown in the lower panels. Yellow arrows point to high-intensity H3p staining indicative of metaphasic cells while red arrow points to lower intensity H3p staining indicative of prophase cells. **C.** Immunofluorescent staining visualizes H3p (red) and 16E2 or GFP (green) in SiHa cells infected with GFP and GFP-16E2 recombinant adenoviruse, the cells were seeded on coverslips and harvested 22 hours post thymidine release. Yellow arrows: metaphasic nuclei, red arrows: prophasic nuclei. Scale bar = 50 μm. **D.** Flow cytometric analysis of A549 cells infected with GFP either treated (Noco) or not by nocodazole, or GFP-16E2 recombinant adenoviruses in synchronized cells 16 h after release from thymidine block. DNA content (blue) and H3p (red) were co-labeled (NT = not transduced). H3p staining density plots were done on the same samples and are shown in the lower panels together with percentage of cells in mitosis. Black arrow: high-intensity H3p staining indicative of metaphasic cells, red arrow: lower intensity H3p staining indicative of prophase cells. **E.** Confocal microscopy visualizes H3p (red) and 16E2 (green) in A549 cells. Yellow arrows: metaphasic cell, red arrows: prophasic nuclei. Scale bar = 10 μm. **F.** Time-lapse microscopy was performed to observe cell cycle progression in synchronized A549 cells infected with GFP- or GFP-16E2-recombinant adenoviruses. Typical mitotic division for GFP-expressing cells became evident 12–13 h after thymidine release. E2-expressing cells do not divide within the 29 h of observation.

Flow cytometric analysis of cell cycle distribution revealed that 6 hours post thymidine release, 78–86% of cells infected with GFP, GFP-16E2, GFP-TAD, and GFP-DBD moved to the next cell cycle phases S and G2/M (Figure 1A, upper panel), the protein levels of transduced proteins, GFP, GFP-16E2, GFP-TAD and GFP-DBD were measured by Western blot at that time point (Figure 1A, lower panel). Within 22 h of thymidine release, the proportion of G2/M population is higher in the E2 expressing cells (44.3%) compared to GFP control cells (20.6%), even higher than TAD expressing cells (27.2%) indicative of a potential cell cycle arrest in G2/M by E2 independently of the expression of the endogenous E6E7 that should be repressed by the full length protein and not by TAD. Increased S phase in GFP control cells indicates the start of second round of cell cycle through high expression of E6E7. Interestingly, the DBD infected cells exhibited a marked G1 arrest as a consequence of repression of E6E7 transcription with no expression of the E2 TAD functional domain, as expected from previous reports [20]. To further define the effect of E2 on host cell cycle we measured the proportion of infected cells in the G2/M 4N peak that were undergoing mitosis using an antibody specific for the phosphorylated serine 10 of histone 3 (H3p), which is only present in mitotic chromatin and is a marker of DNA condensation [21–23]. However, when the intensity of H3p labeling was measured by flow cytometry it appeared less intense in E2 infected cells compared to GFP, GFP-TAD and GFP-DBD infected cells (Figure 1B, lower panel, yellow arrows compared to red arrow), leading us to ask whether full-length E2 induced arrest at an earlier stage of mitosis that was barely detectable in the other conditions. Indeed, immunofluorescent imaging confirmed that most E2-expressing cells were H3p positive but that the signal was primarily distributed as punctate signals in intact nuclei, indicative of prophase (Figure 1C, red arrows), while in GFP control infection, only few cells exhibited strong, uniform H3p expression characteristic of metaphasic cells (Figure 1C, yellow arrows).

We then decided to analyze the modulation of cell cycle by E2 in the A549 human lung carcinoma cells that are HPV negative and retain wild-type p53, which is similarly present in most cervical carcinomas [24]. When we synchronized infected GFP and GFP16E2 A549 cells at the G1/S boundary by thymidine treatment, flow cytometric analysis of cell cycle distribution revealed that, while most of the cells infected with the GFP negative control virus had returned to G1 phase within 16 h of thymidine release, more than 50% of the HPV16 E2-expressing cells had arrested in the G2/M phase of the cell cycle (Figure 1D, DAPI). A similar G2/M arrest was also observed in GFP-TAD but not in GFP-DBD infected A549 cells (Figure S1), indicating that TAD plays a major role in the HPV-16E2 induced G2/M arrest. For comparison, the GFP infected cells arrested in metaphase by nocodazole treatment showed a higher percentage of cells arrested in G2/M and moreover a more intense H3p labeling than E2 arrested cells indicative of an E2-mediated arrest in an earlier phase of mitosis (Figure 1D, compare GFP Noco and GFP-16E2). Immunofluorescent imaging confirmed that most E2-expressing cells were H3p positive and that the signal was distributed as dots in intact nuclei, indicative of prophase (Figure 1E, red arrows), while GFP-infected cells exhibited the strong, uniform H3p expression characteristic of metaphasic cells (Figure 1E yellow arrow). Time-lapse microscopy further demonstrates that E2-expressing A549 cells could not progress to metaphase over 30 h post release while control cells proceeded to mitotic exit within 13 h (Figure 1F and Figure S2). Consistent with these distinct morphologies, when E2-arrested cells were labeled for β-tubulin, a concentric organization of filaments around intact nuclei was evident, whereas control cells displayed a disperse filament arrangement (Figure S3). Taken together, the above data demonstrate that HPV16-E2 induces a durable block in the cell cycle at prophase in SiHa and A549 cells *in vitro*.

### DNA damage response is activated in cells undergoing E2-induced prophase arrest

**Figure 2:**
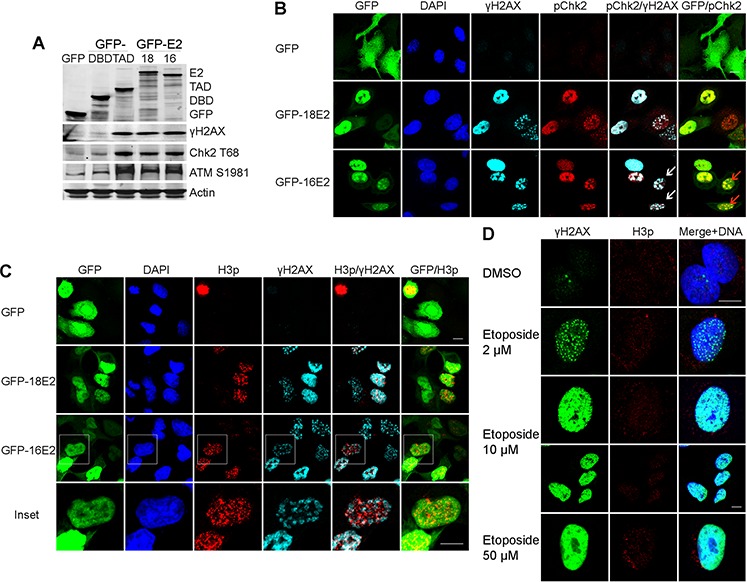
E2 induces a DNA damage signal in A549 cells arrested in prophase **A.** Western blot analyses of lysates from A549 cells expressing GFP-16E2, GFP-18E2, HPV18E2 transactivation domain (GFP-18E2TAD) or the DNA binding domain (GFP-18E2DBD), probed with antibodies against GFP, γH2AX, Chk2^T68^ and ATM^S1981^. **B.** Confocal microscopy of E2-expressing A549 cells double labeled with anti-γH2AX (light blue) and anti-Chk2^T68^ (red). White arrows: co-localization of the DDR markers, red arrows: DDR marker partial co-localization with 16E2 in infected nuclei. **C.** As in (B) except the cells were co-labeled with anti-H3p (red) and anti-γH2AX (light blue). Inset show higher magnification of double stained infected nuclei with distinct punctate signals for the H3p and γH2AX markers **D.** Expression of H3p (red) and γH2AX (green) in A549 cells treated with increasing doses of etoposide. Scale bar = 10μm.

As mitotic entry has been shown to be associated with ATM and DDR activation [16], we postulated that E2 might interfere with the ATM/DDR pathway during the E2-induced prophase arrest in A549 cells. Following phosphorylation of ATM at serine 1981 (ATM^S1981^), activation of the DDR signal is marked by phosphorylation of the chromatin histone H2AX at S139 (γH2AX) and phosphorylation of Checkpoint kinase 2 at threonine 68 (Chk2^T68^). We therefore carried out western blotting to detect these 3 key proteins in A549 cells infected with either GFP, GFP-16E2, GFP-18E2, GFP-18DBD or GFP-18TAD [18]. Expression of full-length E2 from either of the high-risk HPV types (16E2 and 18E2) and the HPV 18E2 TAD only induced ATM phosphorylation and activated the DDR signals, as indicated by increased abundance of ATM^S1981^, γH2AX and Chk2^T68^ proteins compared to control cells infected by GFP or the GFP-E2DBD (Figure 2A). Immunofluorescent labeling revealed that the two DDR activation-associated proteins γH2AX and Chk2^T68^ co-localized as characteristic foci in most of both HPV16 and HPV18 E2-positive nuclei (Figure 2B).

To understand whether the observed DDR induction was associated with cell cycle prophase arrest, we performed co-labeling for H3p and γH2AX in cells that were expressing the E2 protein of either HPV16 or HPV18. Both H3p and γH2AX were expressed as distinct foci in the same E2-positive nuclei of the transduced cells (Figure 2C). These experiments clearly indicated that E2-induced prophase arrest is linked to activation of the host cell DDR signal.

When we used the DNA-damaging agent etoposide to induce the DDR in synchronized A549 cells, we observed either discrete foci or pan-nuclear distribution of γH2AX, dependent on the concentration of etoposide used (Figure 2D), whereas H3p labeling was undetectable. Indeed, rather than exhibiting prophase arrest, A549 cells subjected to etoposide-induced DNA damage were arrested in interphase, as previously reported [25, 26]. Taken together, these data indicate that E2-induced DDR activation occurs together with prophase arrest and is probably independent of DNA damage as DNA damage usually induces cell cycle arrest in interphase. High-risk E2 proteins exert pivotal roles in transcriptional regulation and in viral DNA replication, the modification of H2AX independent of DNA damage may contribute towards creating specific chromatin structure frame allowing ‘non canonical’ functions to be carried out which would need further investigation.

### Strong and persistent activation of Cdc25C^T48^ phosphatase occurred in E2-expressing prophase cells followed by DNA damage response

**Figure 3:**
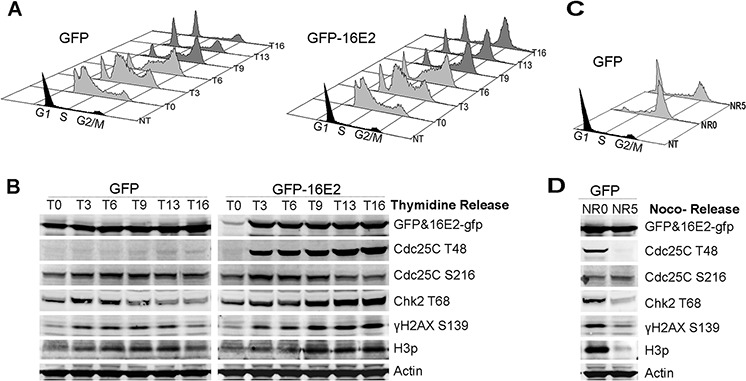
Expression of E2 induces the mitotic activator Cdc25CT48 and coincides with the induction of DDR markers **A.** Thymidine synchronized A549 cells were infected with GFP or GFP-16E2 and released at various time points between 0–16 h, as indicated (T0-T16) for cell cycle analyses by flow cytometry. **B.** Western blot detection of protein levels of the same samples at the same time points. The activated cell cycle regulators Cdc25C^T48^ and H3p were used as markers of mitotic entry. Chk2^T68^ and γH2AX were used as markers of DDR signal activation. **C.** Thymidine synchronized A549 cells infected with GFP were treated by nocodazole for 16 hours (NR0) and were then released from nocodazole for 5 hours and harvested (NR5) for cell cycle analysis by flow cytometry. **D.** Western blot detection of the same proteins as in (B) as indicated.

We next sought to define the causal relationship between E2-induced prophase arrest and DDR activation. Mitotic entry is orchestrated by a complex interplay of factors culminating in the activation of Cdc25C which is phosphorylated on at least 6 residues in order to induce normal mitotic entry in mammalian cells. One of these phosphorylated residue is threonine-48 (Cdc25C^T48^) which is used here as a marker of Cdc25C activation. Conversely, negative regulation of Cdc25C occurs following its phosphorylation on Serine-216 (Cdc25C^S216^), which blocks mitotic entry under normal conditions and after DNA damage [27]. We used flow cytometry to follow cell cycle stable arrest in G2/M of thymidine synchronized A549 cells expressing HPV16-E2 (Figure 3A). The same cells were processed for western blot analyses of E2 and expression of mitotic and DDR markers at various time points after thymidine release (Figure 3B). Maximal E2 expression at around 3 h post thymidine release coincided with a striking activation of mitotic Cdc25C^T48^ (Figure 3B), strongly suggesting that this activation was driven by E2. In comparison, cells arrested in pro-metaphase by nocodazole treatment also expressed Cdc25C^T48^ albeit at a lower level than in E2-induced prophase arrested cells, and this activation was transient as Cdc25C^T48^ disappeared 5 hours post nocodazole release (Figure 3D NR0 indicates nocodazole blocking and NR5 indicates 5 hours post release from nocodazole blocking). This accumulation of Cdc25C^T48^ was accompanied by decreased Cdc25C^S216^ expression, consistent with M phase entry (Figure 3B). Expression of the DDR activation markers Chk2^T68^ and γH2AX in control cells was transient, occurring between 3 and 6 h post release coincident with the S phase seen in flow cytometry (Figure 3A left panel), whereas their increased expression in E2-expressing cells started at 3 h post release similarly to GFP infected control cells but continued to increase and stabilize after 9 h post-thymidine release until 16 h post release which coincides with G2/M arrest as indicated in flow cytometry. This marked stabilization of Chk2^T68^ and γH2AX together after activated Cdc25C indicated that the induction of DDR occurs during normal transition from G2 to mitosis and that it is stabilized in E2 expressing cells due to E2-induced prophase arrest. This was compared to nocodazole treatment which arrests most of the treated cells in mitosis (Figure 3C NR0) and induces a concomitant DDR signal activation (Figure 3D NR0), with a huge accumulation of H3p that indicates an arrest in early metaphase compared to relatively lower accumulation of H3p in E2-expressing cells indicative of prophase arrest, which is consistent with our H3p immunofluorescent staining (Figure 1C, 1E). However, when the cells were released from nocodazole treatment for 5 hours and ∼ 80% of the cells were back to G1 (Figure 3C NR5), specific labeling of mitotic and DDR signals were greatly decreased (Figure 3D NR5) in stark contrast with the persistent signals in E2 expressing cells. We concluded from these experiments that E2-induced prophase arrest is linked to a rapid accumulation of activated Cdc25C^T48^ followed by sustained overexpression of an activated DDR signal.

The M-phase entry with extremely high Cdc25C^T48^ in HPV-16E2 expressing cells may indicate abnormal/premature prophase which could be the reason for the occurrence of DNA damage response in prophase induced by HPV-16E2. E2 may bind to DNA repair components and affect their functions which further amplify DNA damage response. We harvested 16 h post-thymidine release A549 cells expressing GFP or HPV16-E2 to detect whether HPV-16E2 interacts with Chk2^T68^ in G2/M arrested cell population. First, we measured the DNA content and Chk2^T68^ distribution in E2-expressing cells by flow cytometry. Detection of Chk2^T68^ coincided with the peak of 4N DNA content in E2-expressing cells and G2/M arrested cell population and Chk2^T68^ level were more prominent in cells highly expressing E2 (Figure S4A, comparing E2 m.o.i. 100 to E2 m.o.i. 25). Immunoprecipitation experiments indicated that HPV-16E2 can bind to Chk2^T68^ on a dosage dependent manner (Figure S4B), together with the co-localization of HPV-16E2 with Chk2^T68^ (Figure 2B, red arrows), these results further corroborate the link between E2-induced prophase arrest and DDR activation.

### Activation of ATM and CHK2 transcripts in HPV16-associated CIN3 lesions *in vivo*

**Figure 4:**
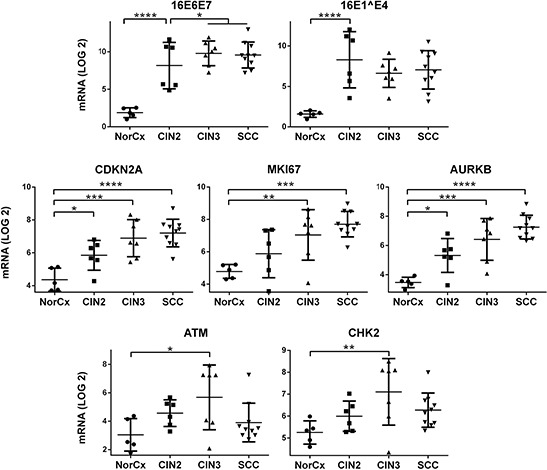
Transcripts of DDR components are less abundant in SCC than in CIN3 samples Digital quantification of viral and cellular mRNAs was performed by NanoString analysis in *n* = 28 microdissected cervical lesions, including samples from normal cervix (NorCx) and HPV16-associated CIN2, CIN3 and SCC lesions. Quantified viral genes were 16E6E7 and 16E1^E4, while the cellular gene transcripts analyzed included the CDKN2A (p16^INK4^) surrogate marker of E7 expression, proliferative marker MKI67, mitotic gene AURKB, and DDR components ATM and CHK2. For each category of lesions the transcript levels were plotted after log 2 transformation. The indicated *p*-values were calculated by 1-way ANOVA. The horizontal bars represent the mean values for each group.

**Figure 5:**
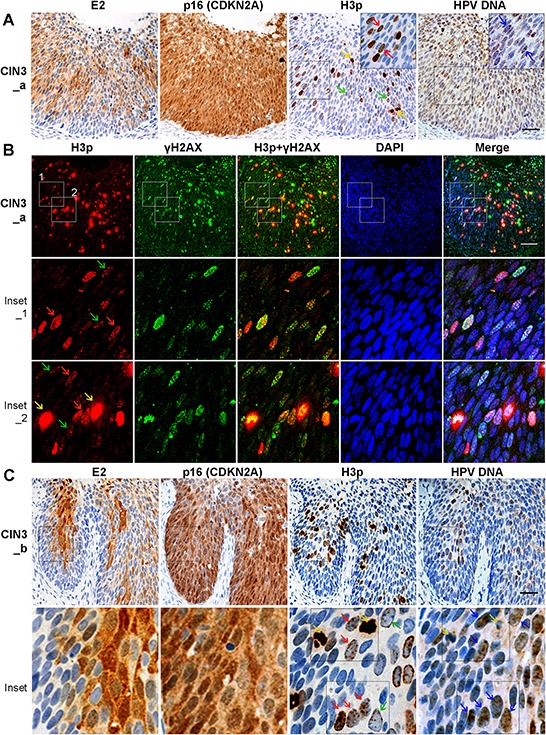
Prophase cells are more common in the areas of HPV16-associated CIN3 lesions that co-express E2 and p16 **A.** Prophase arrest in CIN3 with E2 and p16 co-expression. Consecutive sections of CIN3 lesions were labeled for 16E2, p16 ^INK4^ and, H3p. In E2-positive regions, prophase cells were abundant. HPV DNA was detected by amplified-ISH. **B.** Co-labeling of the mitotic marker H3p (red) and DDR marker γH2AX (green) in CIN3 tissue samples. **C.** Similar as (A) another lesion with early M phase arrest in CIN3 with E2 and p16 co-expression. High magnification images in Inset illustrate that prophase cells host low levels of HPV DNA replication. Yellow arrows: metaphasic cells; red arrows: prophasic cells; blue arrows: HPV DNA. Scale bar = 50 μm.

Having identified a novel role for E2 in inducing a DDR signal *in vitro* together with a cell cycle prophase arrest, we sought to understand the clinical relevance of these findings. We therefore analyzed the levels of transcripts of various markers of cell cycle and DDR in patient samples of HPV16-associated pre-malignant and SCC lesions. We used the NanoString technology to quantify viral and cellular transcripts in microdissected cervical lesions (*n* = 5 normal cervices, *n* = 6 HPV16-associated CIN2 lesions, *n* = 7 CIN3 lesions, and *n* = 10 cervical SCC lesions) (Figure 4). We have previously shown that the presence of E2 transcripts does not always correlate with expression of the corresponding protein, especially in high grade lesions where E2 transcripts might be abundant with no expression of the protein [19, 28]. Transcript quantification indicated that in the majority of cases, E6E7 transcripts were more abundant in SCC and CIN3 lesions than in CIN2 lesions. The reverse trend was observed for E1^E4 transcript levels, which are disrupted during integration of the viral genome in the cellular genome and were typically less frequent in CIN3 and SCC than in CIN2 lesions. We also observed a significant increase in the expression of cellular proliferative and mitotic markers including CDKN2A, MKI67 and AURKB in CIN and SCC lesions when compared with samples from normal cervix (Dunnetts multiple comparison *p*-values < 0.05). As expected, expression of these 3 key pro-proliferative genes was progressively increased in more clinically-advanced lesions (linear trend slope for CDKN2A = 0.67, MKI67 = 0.50, and for AURKB = 0.48; *p* < 0.0001). We also assessed the transcript levels of 2 representative DDR genes and observed that transcripts of ATM and CHK2 were similarly more abundant in HPV16 lesions compared with normal tissue. These genes were significantly over-expressed in CIN3 lesions (*t*-test ATM *p* = 0.040 and CHK2 *p* = 0.027) and ATM was also over-expressed in CIN2 (*p* = 0.038). Interestingly, expression of DDR genes was lower in SCC lesions than in CIN3. The significant increased expression of DDR genes in CIN3 lesions compared to SCC and CIN2, supports the conclusion that E2 might play a role in DDR activation in clinical samples as the E2 protein is expressed in CIN3 (Figure 5A, 5C), albeit at relatively lower level than in lower grade lesions, [6, 19].

### Concomitant E2 and DDR markers expression is found together with low levels of viral DNA in HPV16-associated CIN3

We next investigated whether the DDR activation and nuclear expression of prophase H3p, that we observed in E2-expressing cells *in vitro*, was also paralleled in CIN3 lesions from patients. We employed IHC to detect E2, p16^INK4^ (CDKN2A, a surrogate marker of E7 expression) and H3p, alongside immunofluorescent labeling for H3p and γH2AX. E2 was found expressed with p16 proteins in the intermediate and upper layers of a sub-population of CIN3 lesions (2 out of 7 CIN3 cases) (Figure 5A, 5C). Labeling of serial sections with the same H3p-specific antibody for both IHC and IF revealed a predominance of the H3p labeling pattern characteristic of prophase, specifically multiple discrete dots in intact nuclei (prophase: red and green arrows) (Figure 5; Figure S5; H3P). Metaphase (yellow arrows) cells were also observed. Simultaneously, we detected HPV DNA by amplified-ISH in adjacent tissue sections and observed that low levels of viral DNA were evident alongside H3p expression, where E2 and p16 were also co-expressed (Figure 5A, 5C, inset, blue arrows). High magnification images in Figure 5C illustrate the prophase H3p phenotype and HPV DNA within the same cells. Furthermore, co-labeling of γH2AX and prophase H3p in intact nuclei was detected in 20 to 40% of cells in the intermediate and upper layers of the two CIN3 samples presented, whereas only 1–2% of these cells are obviously in metaphase with a very strong H3p signal as in Figure 5B inset 2 (shown by yellow arrows) (Figure 5B; Figure S5). These data indicate that the DDR signal is induced in mitotic cells *in vivo*, most often in cells of the intermediate and upper layers of CIN3 lesions where both E2 and p16 are co-expressed and low levels of HPV DNA is detected. The specification of these locations where cells frequently co-express E2 and the E7 surrogate marker p16^INK4^ is consistent with a requirement for E7 in eliciting the mitotic entry and for E2 in arresting cells in prophase which was associated with a low level of viral DNA. This low level of viral DNA could be viral replication intermediates not fully replicated or not resolved.

**Figure 6:**
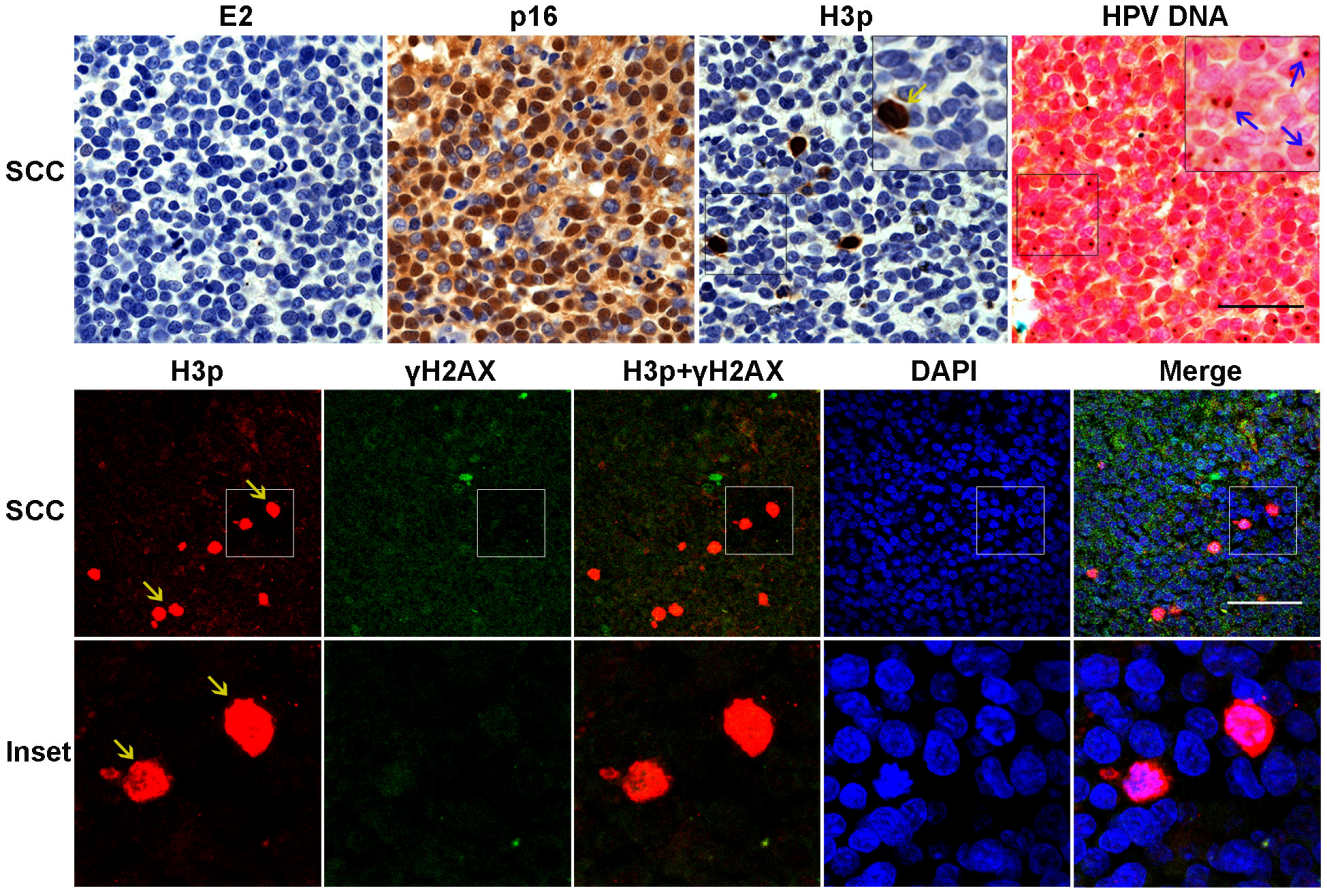
Metaphase but not prophase patterns of H3p expression in HPV16 associated SCC lesions Serial sections from SCC samples were labeled for 16E2, p16 ^INK4^, H3p, and HPV DNA. High expression of p16^INK4^ was observed in the absence of E2, while amplified-ISH of the contiguous section shows integration of HPV DNA visualized as punctate brown signals (Blue arrow). H3p stained by IHC or IF staining shows mostly metaphasic cells (yellow arrows). Lower panel shows co-labeling for H3p (red) and γH2AX (green). Scale bar = 50μm.

We next assessed H3p expression together with the distribution of E2, p16^INK4^, and viral DNA in HPV16-associated SCC lesions. In agreement with our previous findings [6, 19, 28], we observed that E2 protein was undetectable from the majority of SCC samples, whereas p16^INK4^ was highly expressed in these lesions (Figure 6). H3p-positive cells were detected in SCC, although in lesser number than in CIN3 (∼ 0.5%), but unlike the dominant prophase pattern of H3p expression in CIN3 lesions, the expression pattern in SCC was predominantly in metaphase (Figure 6, yellow arrows). In addition, detection of γH2AX labeling was uncommon in SCC cells and did not coincide with H3p-positive signal. Crucially, amplified-ISH confirmed that no detectable replication of episomal HPV DNA was occurring in the SCC lesion and that viral DNA had been successfully integrated in the cellular genomes (Figure 6 upper panel right).

Taken together, these data strongly suggest that the increased proportion of cells in prophase in CIN3 depends on E2 expression and that expression of E2 either directly or indirectly (through the cell cycle arrest) induces activation of a strong DDR signal in the same cells. It should be noted however that E2 alone is not an inducer of the cell cycle contrary to E7 which is the main inducer of cell cycle in HPV infected lesions. Interestingly both viral proteins seem to be expressed in the CIN3 lesions where the DDR and the mitotic signals are detected thus suggesting concomitant functions of the two viral proteins in the control of cell cycle and DDR activation *in vivo*. The exact role of E7 in the *in vivo* situation is however difficult to assess although it is clear from our experiments that lesions expressing E7 and not E2, as in SCCs, do not exhibit the cell cycle arrest linked DDR signal.

## DISCUSSION

In the present study we demonstrated that HPV-E2 protein induces a prolonged host cell cycle arrest in prophase and elicits a DDR signal *in vitro* in cell lines independent of their association with HPV. Similarly, in patient's samples, expression of E2 coincides with activation of markers of both mitosis and DDR signal associated with low levels of viral DNA replication mainly seen in HPV16-associated pre-malignant CIN3 lesions of the cervix. We previously identified a potential mechanism by which E2 could induce cell cycle mitotic arrest *in vitro*; E2 protein inactivates the mitotic ubiquitin ligase APC/C and supports the accumulation of mitotic regulators such as CyclinB1 [13]. Furthermore, E2 can interact with elements of the spindle assembly checkpoint to delay its inactivation during mitosis, thus inducing the mitotic arrest described here and in previous reports [13, 14]. In the current report, we demonstrate that the activation of host cell DDR signal by HPV16-E2 occurs concomitantly with M-phase entry and is maintained during the prolonged E2-induced prophase arrest *in vitro*.

The main aim of the current study was to assess the putative role played by E2 in activating the host cell DDR signal. DDR signal has recently been shown to be part of the mitotic process independent of DNA break, as playing a role in the spindle assembly check point [29]. Interestingly, both mitotic arrest and DDR response were found to be quite sustained in E2-expressing cells in our *in vitro* experiments and we found that markers of mitotic arrest as well as DDR signal where also detected *in vivo* in patient samples expressing E2. Our analyses revealed that markers of prophase as well as markers of activated DDR response can occur within the same cells in some HPV16-induced CIN3 lesions. Furthermore, detection of key markers of these processes coincides with low levels of viral DNA replication. Viral replication in CIN3 is usually at low levels, similar to that seen in the parabasal layers of low grade CIN, where viral amplification is initiated but only completed following the differentiation of the epithelium.

Presence of low levels of viral DNA replication however is in line with recent reports showing that viral DNA replication can use the elements of the DDR machinery instead of the cellular replication factors. This hypothesis thus suggests that viral DNA replication actually does not take place in S-phase but rather in more advanced stages of the cell cycle as in prophase for instance. Early mitosis is also accompanied by a clear activation of the DDR thus allowing the best conditions for viral DNA replication to occur. In this hypothesis, it is clear that the E2 viral protein could play determinant roles, not only to recruit the E1 helicase to the origin of replication, but also to set up the recipient cells in the right state for viral DNA replication. In this regard, however, and as the viral DNA replication and E2 expression are optimal in lower grade CIN [6], the same phenomenon should take place in CIN1 and CIN2 lesions. We could not detect mitotic or DDR signals in E2 and viral DNA positive cells in lower grade CIN and this probably indicates that the viral DNA replication mechanisms are different in these low grade differentiated lesions. Interestingly, E7 has been shown to be able to activate the ATM DNA damage pathway [12] thus suggesting that it might also play a role in the instability detected in CIN3 lesions where both E7 and E2 are expressed. As mentioned earlier, while E2 is a strong modulator of cell cycle on its own, it is not able to induce cell cycle entry or reactivate the cell cycle in arrested cells. This activity is on the contrary well controlled by E7 which is not expressed at high levels in lower grade CIN [6, 28, 30]. As in the low grade lesions the majority of cells expressing E2 are not cycling, they probably are not modified by expression of the viral protein. It is worth however discussing the possibility that E2 could be expressed at low levels in cells that are expressing also E7 in the parabasal layers of CIN2 for example, and that, in at least some of these cells, viral DNA replication can be initiated through activation of the DDR machinery as suggested in the intermediate layers of CIN3. This phenomenon could also be too transient to be detected by the technology used here, potentially requiring the analysis of more clinical samples.

Our observations therefore suggest that when E2 is expressed, even at relatively low levels, in cells that have re-entered the cell cycle, it may play a crucial role in maintaining the cells in a state compatible with viral DNA replication. In addition, as this specific situation is found in CIN3, it leads to the exciting hypothesis that E2 could participate in the carcinogenic progression by favoring integration of part of the viral genome in the cellular genome as both mitotic arrest and genomic instability are favoring this event, together with the presence of freshly replicated viral DNA. Therefore, it appears that the main changes occurring during the transition from CIN3 to SCC are also dependent on E2 expression: a certain level of co-expression of E2 and E7 oncoprotein in CIN3 is followed by disruption of E2 protein expression and overexpression of E7 in SCC with decreased activation of the DDR signal from CIN3 to SCC and switching from low levels of viral DNA replication in CIN3 to viral genomic integration in SCC. These shifts from CIN3 to SCC are in part controlled by E2 and confirm E2 as a player in inducing genomic instability and promoting carcinogenesis in HPV-associated cervical lesions.

## MATERIALS AND METHODS

### Tissue specimens

A total of 130 formalin-fixed and paraffin-embedded (FFPE) cervical punch biopsies, tissue samples obtained by loop electrosurgical excision procedure and cold knife cervical conization were obtained from department of pathology, KK hospital and National University Hospital, Singapore. The specimens included 10 normal cervical tissues, *n* = 40 CIN1, *n* = 40 CIN2, *n* = 40 CIN3 and *n* = 40 SCC samples, of which *n* = 6 CIN2, *n* = 7 CIN3, *n* = 10 SCC were selected for further study based on their positive HPV16 association (alongside *n* = 5 normal cervices). Classification of specimens was validated by 2 pathologists. The study was approved by the institutional review board of SingHealth of Singapore (CIRB & DSRB approval No. 205-001) and National University of Singapore (NUS-IRB approval no. NUS827).

### HPV genotyping

DNA extraction was carried out using the QIAamp DNA FFPE Tissue Kit (QIAGEN, Germany). An aliquot of 100 ng total extracted DNA was used for HPV genotyping with the HPV GenoArray test kit according to the manufacturer's instructions (Hybribio Limited, Hong Kong). This approach allowed us to identify 21 distinct HPV genotypes, including 5 low-risk types (types 6, 11, 42, 43, and 44), 14 high-risk types (16, 18, 31, 33, 35, 39, 45, 51, 52, 56, 58, 59, 66, and 68), and 2 intermediate-risk types (CP8304 and 53).

### RNA extraction and gene expression quantification

Total RNA was extracted from FFPE samples using the Qiagen RNeasy FFPE kit. Gene expression was profiled using the NanoString nCounter Analysis System according to the manufacturer's instructions. The detailed methods were described previously [19]. Probe sets specific for the HPV16 viral genes E2, E1^E4, E6E7, and the cellular genes CDKN2A (p16), MKI67, AURKB, ATM and CHK2 were designed and synthesized by NanoString Technologies (Supplementary Table S1).

### Cell culture, viral infections and time-lapse microscopy

SiHa and A549 cells were grown in Dulbecco's modified Eagle Medium (DMEM) supplemented with 10% fetal bovine serum (FBS) and 1% penicillin-streptomycin, in a humidified incubator at 37°C, 5% CO_2_. Cell infection with recombinant adenoviruses expressing the green fluorescent protein (GFP) (expressing either GFP only or GFP fusion proteins) was conducted at m.o.i. of 50 to 100, as previously described [13]. For live microscopy, images were captured every 30 min in phase contrast and fluorescence using a 20X objective with the Applied Precision Delta Vision Deconvolution microscopy system at 37°C and 5% CO_2_. Images were then processed with softWoRx software and converted into Adobe Photoshop.

### Cell synchronization and flow cytometry

We used thymidine double blocks to synchronize HPV16 positive cervical carcinoma cell line (SiHa): after a first 24 hours thymidine (2.5 mM) block, the cells were released for 2 hours and infected with the recombinant adenoviruses expressing GFP-16E2, GFP-TAD GFP-DBD and GFP as control, followed by a second thymidine block. The cells were released 22 h later for cell cycle analyses.

A549 cells were treated with 2.5 mM thymidine for 24 h and infected with recombinant adenoviruses expressing GFP or the GFP-16E2 fusion protein half way through the thymidine treatment. After which the cells were released from thymidine block and harvested for analysis at the indicated time points (from T0 to T16). For synchronization in metaphase, cells were treated with thymidine for 24 h and then released for 16 h in the presence of 100 ng/ml nocodazole (NR0), nocodazole was then removed and cells were harvested 5 hours later (NR5 corresponding to 5 h post nocodazole release) for cell cycle analysis. The cells were fixed in 70% ethanol before flow cytometry to detect DNA content using 0.5 μg/ml DAPI and a 1:500 dilution of an anti-H3p antibody (Ab7031, Abcam). Cell cycle analyses were carried out using an LSR flow cytometer (BD Biosciences, USA).

### Immunofluorescence

Tissue sections or SiHa and A549 cells grown on coverslips were permeabilized in 0.1% Triton and incubated with mouse anti-γH2AX antibody and rabbit antibodies against H3p, β-tubulin, and Chk2^T68^ (listed in Supplementary Table S2). Secondary labeling employed anti-rabbit and anti-mouse antibodies conjugated to Alexa Fluor® 488, 568 or 647, and nuclei were counterstained with DAPI. Images were observed using an Olympus FV1000 upright confocal microscope or Zeiss AxioImager upright microscope and captured at 405 nm, 488 nm, 568 nm and 647 nm using 40x objective lens, before processing with FV10-ASW 3.0 Viewer or AxioVision software.

### Western blot, immunoprecipitation

Cells infected with GFP, GFP-16E2, GFP-DBD or GFP-TAD recombinant adenoviruses harvested in different conditions were used for protein extraction. Proteins were extracted with an extraction buffer (300 mM NaCl, 0.5% Nonidet P-40, 50 mM Tris-HCl with pH8.0, 1 mM EDTA, protease and phosphatase inhibitors) for 30 min at 4°C, followed by centrifugation at 13000 rpm for 30 min. Proteins were separated by Sodium Dodecyl Sulfate-PolyAcrylamide Gel Electrophoresis (SDS-PAGE) and immunoblotting experiments were conducted using anti-GFP, anti-γH2AX, anti-Chk2^T68^, anti-ATM^S1981^, anti-Actin, anti-Cdc25C^T48^, anti-Cdc25C^S216^, and anti-H3p antibodies (listed in Supplementary Table S2). Extracts containing 1 mg of total proteins were immunoprecipitated using GFP-Trap_A beads (ChromoTek) according to the manufacturer's instructions.

### Immunohistochemistry (IHC) and amplified *in situ* hybridization (amplified ISH)

Consecutive sections were labeled for IHC using purified anti-HPV16 E2C [6], anti-p16^INK4^, anti-γH2AX and anti-H3p antibodies (Supplementary Table S2) according to previously published methods [6]. ISH was performed using broad spectrum HPV-biotinylated DNA probe sets (Dako Y1401) to detect low-copy number HPV DNA replication via the GenPoint™ Tyramide Signal Amplification System (amplified-ISH) (Dako K0620) according to the manufacture's guidelines.

### Statistical analyses

Gene expression values were log-transformed before ANOVA using Graphpad software (Prism). Dunnett's correction was used for multiple analyses. The single-df linear trend test was applied following ANOVA where indicated.

## SUPPLEMENTARY FIGURES AND TABLES


