# Supplementary material for: HPV16-E2 induces prophase arrest and activates the cellular DNA damage response in vitro and in precursor lesions of cervical carcinoma
Source: Oncotarget. 2015 Oct 14;6(33):34979–91. doi: 10.18632/oncotarget.5512 (PMC4741503; doi:10.18632/oncotarget.5512)
Supplement: Supplementary file 1 [file oncotarget-06-34979-s001.pdf]

SUPPLEMENTARY FIGURES AND TABLES

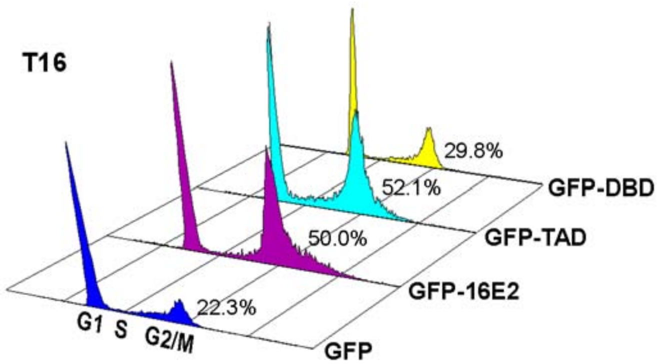

Supplementary Figure S1: Both HPV-16E2 and TAD induce G2M arrest in A549 cells. Thymidine synchronized A549 cells were infected with GFP or GFP-16E2, GFP-TAD and GFP-DBD and released at 16 h time point, the cells were harvested for cell cycle analyses by flow cytometry.

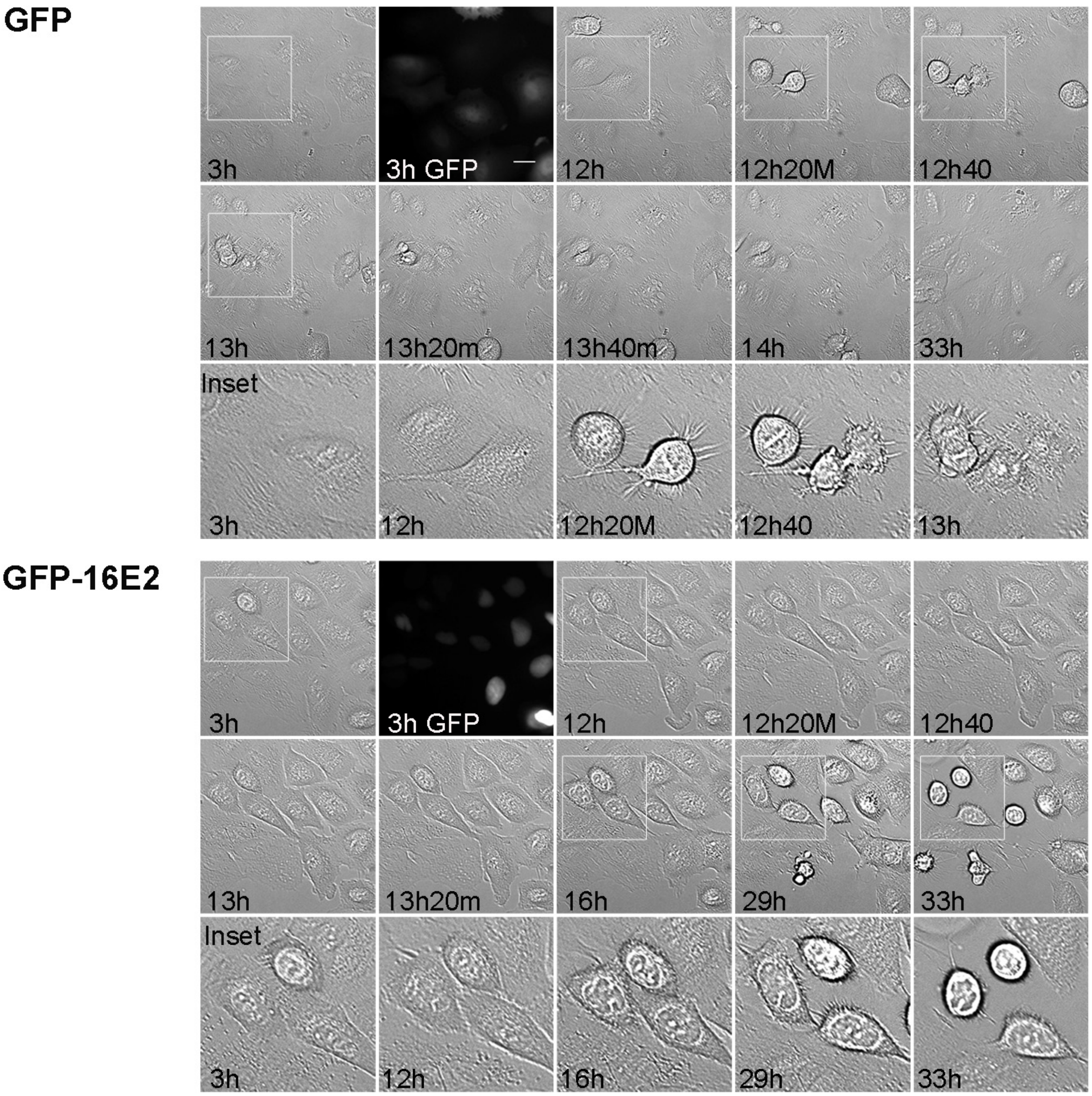

Supplementary Figure S2: Cell cycle division cannot be completed in E2 infected cells. Time-lapse microscopy was performed to observe cell cycle progression in GFP or GFP-16E2 infected cells. Images from 3–33 h after release from the G1/S boundary were captured. Scale bar = 50 μm, labeled in GFP fluorescent image.

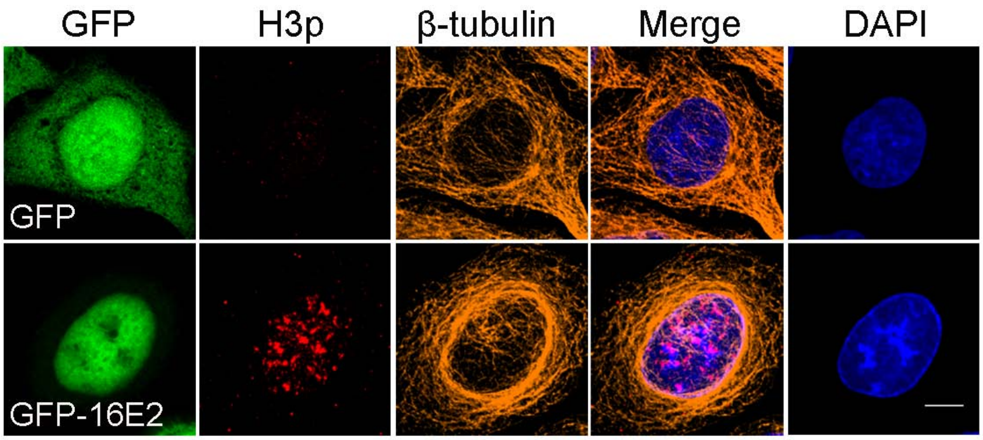

Supplementary Figure S3: β-tubulin disorder in GFP-16E2 arrested A549 cells. Confocal microscopy visualizes H3p (red) and β-tubulin (brown) in GFP and GFP-16E2 infected A549 cells. Scale bar = 10 μm.

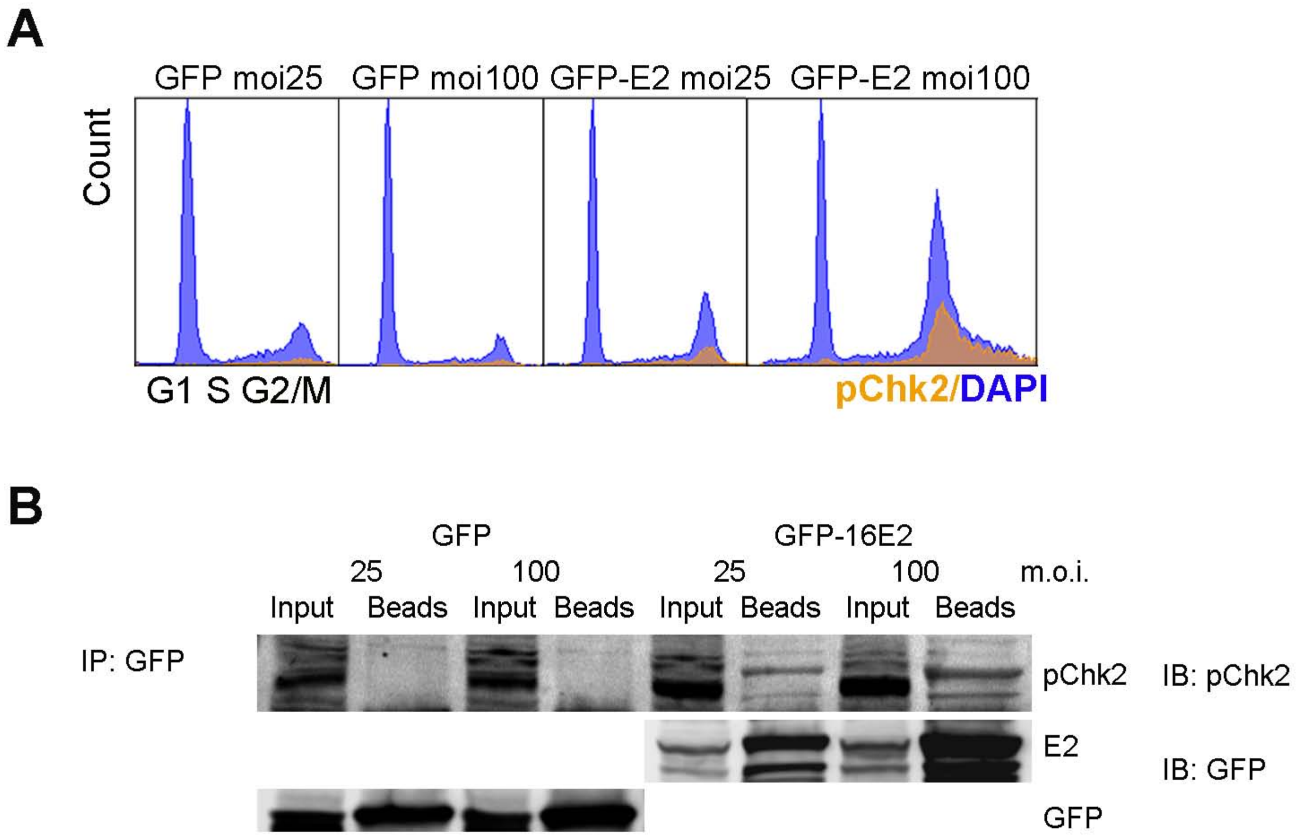

Supplementary Figure S4: GFP-16E2 interacts with endogenous Chk2^{T68}. A. Thymidine synchronized A549 cells were infected with GFP or GFP-16E2 at different m.o.i. and released at 16 h time point for cell cycle analyses by flow cytometry. Marked pChk2 (Chk2^{T68}) expression was observed in the cells within the G2/M 4N peak of E2 infected cells. **B.** Same experiment as in (A) followed by protein extraction. A549 cell extracts were immunoprecipitated against GFP and then analyzed by western blot using the specific Chk2^{T68} antibody. Input controls comprised 2% lysates used for immunoprecipitation.

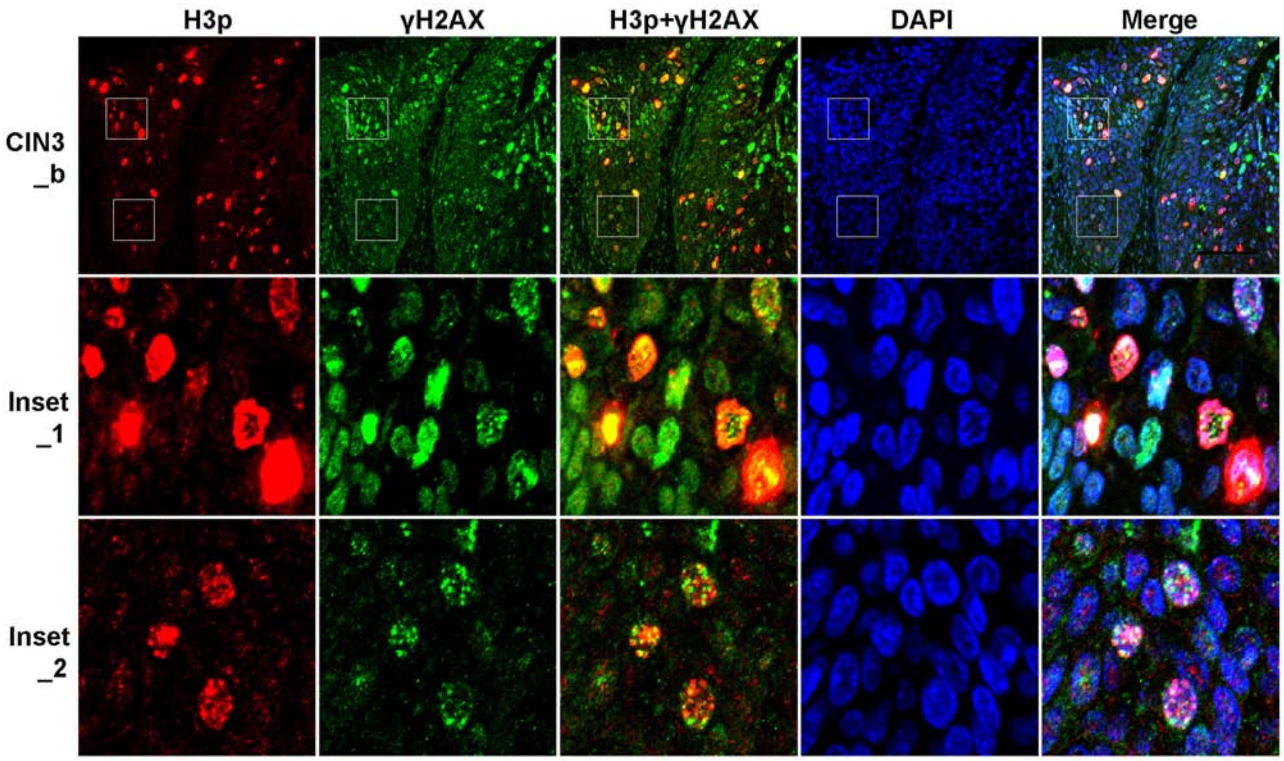

Supplementary Figure S5: DDR activation in prophase cells in another CIN3 lesion. Co-labeling of the mitotic marker H3p (red) and DDR marker γH2AX (green) in CIN3 lesion as shown in Figure 6C on a different patient sample. Scale bar = 50 µm.

Supplementary Table S1: The sequences of the probe sets of the viral and cellular genes for NanoString

Gene	Sequence
HPV16 E1^E4	TGCCCCATCTGTTCTCAGAAACCATAATCTACCATGGCTGATCCTGCAGCAGCAAC<br>GAAGTATCCTCTCCTGAAACTATTAGGCAGCACTTGGCCAACCA
HPV16 E6E7	ATGTCTTGTTGCAGATCATCAAGAACACGTAGAGAAACCCAGCTGTAATCATGC<br>ATGGAGATACACCTACATTGCATGAATATATGTTAGATTTGCAACC
CDKN2A (p16)	AAGCGCACATTCATGTGGGCATTTCTTGCGAGCCTCGCAGCCTCCGGAAGCTGT<br>CGACTTCATGACAAGCATTTTGTGAACTAGGGAAGCTCAGGGGGGT
MKI67	AGCAGATGTAGAGGGAGAACTCTTAGCGTGCAGGAATCTAATGCCATCAGCAGGCA<br>AAGCCATGCACACGCCTAAACCATCAGTAGGTGAAGAGAAAGAC
AURKB (Aurora B)	AGATGCTCTAATGTACTGCCATGGGAAGAAGGTGATTCACAGAGACATAAAGCCA<br>GAAAATCTGCTCTTAGGGCTC
ATM	ACGCTAAGTCGCTGGCCATTGGTGGACATGGCGCAGGCGCGTTTGCTCCGACG<br>GGCCGAATGTTTTGGGGCAGTGT
CHK2	CAGCGTTACCCAGTCCCAAGGCTCCTCCTCACAGTCCCAGGGCATATCCAGCTC<br>CTCTACCAGCACGATGCCAAACT

Supplementary Table S2: Antibody list

Protein	Resource	Clone	Cat No.	Brand	IHC/IF	WB
**HPV16E2**	Ra		Nil	homemade	1:100	
**p16**	Ms	clone JC8	sc-56330	Santa Cruz	1:100	
**H3p(S10)**	Ra		ab7031	Abcam	1:500	1:1000
**γH2AX**	Ms	2F3	NB100–78356	Novus	1:500	1:2000
**Chk2**^{T68}	Ra		#2662	Cell Signaling	1:100	1:1000
**β-tubulin**	Ms	TUB 2.1	T4026	Sigma	1:1000	
**ATM**^{S1981}	Ms	10H11.E12	#4526	Cell Signaling		1:1000
**Cdc25C**^{T48}	Ra		#9527	Cell Signaling		1:1000
**Cdc25C**^{S216}	Ra		#9528	Cell Signaling		1:1000
**GFP**	Ra		TP401	Torrey Pines Biolabs		1:5000
**Actin**	Ra		A2066	Sigma		1:3000
